# Case of Hernia Cerebri

**Published:** 1816-12

**Authors:** T. G. Coombe

**Affiliations:** Member of the Royal College of Surgeons in London, and of the Royal Medical Society in Edinburgh.


### *For the London Medical and Physical Journal.*

*Case of Hernia Cerebri;* by T. G. Coombe, Member of the Royal College of Surgeons in London, and of the Royal Medical Society in Edinburgh.

WILLIAM PLATT, aged fifteen years, received a violent injury in the head, from an accident in a coal-pit. When called to his assistance, I found him labouring under all the symptoms of compressed brain, with a lacerated wound in the scalp, over the left side of the coronal suture. The skull was also fractured, which, on enlarging, the wound appeared to extend, in different directions, over the frontal and left parietal bones: a portion of the former being driven in, had lacerated the dura mater. I removed this depressed portion of bone, as well as several projecting points, with great facility, by means of Hey's saw; and found an opening in the cranium, nearly two inches in diameter. On the following day, he was relieved from every symptom of compression; but, his pulse being accelerated, I took twelve ounces of blood from the arm, and gave him some purging medicine. These had the desired effect, and he was, in every respect, better, though still apparently insensible to surrounding objects. Yet he was easily roused, and gave rational answers to any questions put to him; nor had he any stertorous breathing, or other symptoms of compression. On the fourth day, I removed the dressings, and was pleased to find the wound in a very promising state, having partly united. He was perfectly sensible, and able to take light food; made no particular complaint; nor had he any fever, or other bad symptom. I dressed the wound daily, which continued to look well, until the tenth day, when a small puffy tumour appeared, pushing up the part of the wound which had already united. This tumour continued to increase rapidly, when the cicatrix over it assumed an unhealthy appearance. On the fifteenth day it gave way, and a portion of brain instantly protruded, which, in a very short time, became the size of a hen's egg, covered by a thin vascular membrane, which, of course, was pia mater. From the first appearance of this tumour, which pulsated so forcibly as to be seen with the naked eye, the symptoms of compression returned, and increased in violence, in proportion to the growth of the tumour: the top soon acquired a soft unhealthy appearance, which I removed by means of a common spatula. In a very few hours after, a discharge of thin bloody fluid commenced, which continued for twentyfour hours, to such a degree, that I calculated several ounces of fluid had been discharged. This great evacuation from within the cranium being speedily followed by a cessation of the symptoms of compression, and the boy being again quite sensible, (though dreadfully emaciated,) induced me to augur rather favourably of the case, as I was inclined to think the tumour would eventually, if the boy lived long enough, slough off by small portions,—an event recorded by Mr. Hill in some of his Cases in Surgery. These flattering appearances, however, were of very short duration, for, though he had no return of any symptoms indicating compression of the brain, he evidently sunk very fast, and, on the thirtieth day, died.

*Examination after Death.*—On turning back the integuments, and thereby denuding that part of the cranium in which the injury had been received, I found that the root of the tumour completely occupied the whole of the aperture, which was made by the removal of the depressed portion of bone. The part of the tumour which still remained was of a much firmer consistence than what had been taken away by the spatula. I took hold of it, and easily detached it from its connection with the subjacent brain. I next proceeded to saw through the cranium, and removed one-fourth of the calvaria, on the anterior part of the left side of the head: underneath this was a large quantity of thick dark-coloured fluid, which appeared to consist of blood with brain in a state of suppuration, containing several clots or coagula of blood; and so extensive was the injury which this organ had sustained, that at least one-third of the left hemisphere of the cerebrum was thus destroyed, or diseased; and consequently I was unable to pursue any further anatomical examination.

*Observations.*—I regret that, in laying this case before the public, I cannot offer any opinion as to the real nature of this disease, or propose any treatment promising a successful result. My only wish is to give a clear statement of the symptoms which occurred during life, and the appearances which presented themselves after death, hoping that it will engage the attention, and draw forth some information from a more experienced practitioner. I think this case very strongly corroborates the opinion which Mr. Abernethy, and one or two other authors, have formed as to the nature of this disease. Mr. Abernethy thinks that it proceeds from an injury done to a part of the brain by concussion or contusion, terminating in a diseased state of the vessels, and thereby producing an effusion of blood into the substance of the the brain.* In the case which I have related, there is every reason to believe that considerable injury was done to the brain; and that much internal hæmorrhage took place, which, being at first confined under the tumour, produced apoplectic symptoms, all of which subsided on the discharge which succeeded the removal of the surface of the tumour; and these symptoms never returning, led me to think the patient could not have died from compression, which the dissection clearly proved,—for the diseased state in which I found so great a portion of the brain was sufficient to account for the death of the patient, and excited astonishment in my mind that he had lived so long, though I have seen many instances of the great injury that the brain will sustain without producing immediate death. Mr. Hill states that he has been successful in one or two instances, by putting the point of a lancet through the root of the tumour, and, by that means, from time to time, evacuating any fluid confined beneath; but, in the present case, I cannot think such treatment would have saved the life of the patient, as I do not conceive that he died from compression of the brain, but from the great and deep-seated injury that that organ had sustained.

*Newcastle, Staffordshire;*
*Nov.* 9, 1816.

* See his book on Injuries of the Head.

